# Lesson Learned From Hospital Palliative Care Service in a Cancer Research Center in Italy: Results of 5 Years of Experience

**DOI:** 10.3389/fonc.2022.936795

**Published:** 2022-06-27

**Authors:** Sara Alquati, Carlo Peruselli, Caterina Turrà, Silvia Tanzi

**Affiliations:** ^1^ Palliative Care Unit, Azienda USL – IRCCS di Reggio Emilia, Reggio Emilia, Italy; ^2^ SICP Società Italiana di Cure Palliative, Milano, Italy; ^3^ Department of Hospital Pharmacy, Azienda USL-IRCCS di Reggio Emilia, Reggio Emilia, Italy

**Keywords:** palliative care service, hospital, education, retrospective analysis, quality improvement, cancer

## Abstract

**Background:**

International studies have documented that over a third of all hospital beds are occupied by patients with palliative care needs in their last year of life. Experiences of Palliative Care Services that take place prevalently or exclusively in hospital settings are very few in Italy.

**Objective:**

Describe clinical, educational and research activities performed by a hospital PCS and discussing opportunities and critical issues encountered in an Italian Cancer Center.

**Method:**

Retrospective data regarding adults with advanced stage diseases referred from January 2015 to December 2019.

**Results:**

*Clinical activity -* The PCS performed 2422 initial consultations with an average of 484 initial consultations per year. A majority of patients had advanced cancer, from 85% to 72%, with an average of 2583 total consultations per year and an average of 4.63 consultations per patient. The penetrance has increased over time from 6.3% to 15.75%. *Educational and research activity* - Since 2015, PCS has provided training to health professionals (HPs) of different departments of our hospital. Most of the educational projects for HPs were part of research projects, for example the communication training program, management of pain and end-of-life symptoms and the training program for PC-based skills.

**Conclusion:**

Our data suggests that a PCS able to provide palliative care to inpatients and outpatient and continuous training support to other hospital specialists can relatively quickly improve the level of its penetrance in hospital activities.

## Introduction

Around the world, the demographic, epidemiological and social situation is constantly evolving with a progressive increase of patients suffering from chronic degenerative diseases and palliative care (PC) needs. The prevalence of adult patients with PC needs has been estimated to correspond to 1-1.4% of the European population (1–5) while each year the incidence of adult patients with PC needs in their last period of life has been estimated between 69% and 84% of all deaths/year (6, 7), with forecasts of further growth of these percentages (8). In Italy, the prevalence of adult patients with PC needs can be estimated at 524,000-733,000, while the incidence of patients with PC needs in their last period of life can be estimated at 465,000-517,000 (1).

Many international studies have documented that over a third of all hospital beds are occupied by patients in their last year of life, with palliative care needs that significantly affect their quality of life (9, 10). A large percentage of patients die in the hospital or are admitted to the hospital at least once in the last 6 months of life (11).

The Italian National Institute of Statistics (ISTAT) announced that in 2015, 42.6% of deaths occurred in the hospital, 39.6% at home, 5.7% in a hospice, and 9.2% in nursing homes, with a significant difference in mortality at home between the Italian regions of the Center-North (30.7%) compared to those of the South and the Islands (58.6%) (12).

In Italy, a specific law (13) in 2010 established the right of every citizen to access PC and pain therapy and established that regional and local Palliative Care Networks should be able to provide care to all people with PC needs, regardless of their age, pathology, and care setting. It is a highly innovative law, approved by the Italian Parliament unanimously, and which has received the consensus of public opinion and Italian palliative care professionals.

Unlike in the United States and in Western European countries, where continual growth in the number of Palliative Care Services (PCS) in hospitals has been reported (14–16), PC in Italy has developed from its outset primarily as a home care service and then in dedicated beds in hospices. Currently more than 300 hospices (17) and over 300 home PCSs are operating in Italy, while experiences of PCS that take place prevalently or exclusively in hospital settings are very few (10, 18).

Hospitals’ PCSs are to be considered specialized second-level services, with staff who performs this activity full-time (or in any case as a prevalent activity), and who has completed an advanced theoretical and experiential training approach in the field of palliative medicine. This staff can face complex needs, and can implement training and quality improvement programs in the context in which it operates (19, 20). Studies, mostly performed in the United States, have reported that involvement of hospitals’ PCSs was found to reduce the length of hospital stay, to improve communication regarding care goals, and to effectively improve quality of care for cancer patients, which resulted in a reduction in diagnostic tests, a decreased use of intensive care and less aggressive treatments during the last week of life (21–23).

The aim of this article is to describe clinical, educational and research activities performed by a hospital PCS in Italy and to discuss opportunities and critical issues encountered during PCS experience in an Italian Cancer Research Center.

## PCS History

Our PCS is a specialized hospital-based unit with no dedicated beds in a Reggio Emilia hospital, a 900-bed cancer research hospital. The PCS was established in April 2013 as a part of a research project on implementation of early PC intervention in hospital for advanced cancer patients (24). At present, the PCS staff includes three senior physicians and two advance practice nurses, dedicated full-time to hospital palliative care (25), an advance practice nurse expert in training and a data manager dedicated to the collection and analysis of research project data.

The PCS assists outpatients and inpatients with advance oncological disease or chronic progressive illnesses. A psychologist is also involved in bi-weekly team meetings and in the care of patients and relatives with severe psychological suffering. PCS is very often involved in clinical situations and treatment pathways concerning end of life care that require ethical clarification (26). In these cases, the hospital’s bioethicist is also called to attend team meetings to consult and discuss the best management of a specific case.

The vision of the PCS takes as reference the definition of palliative care proposed by the WHO in 2002 (27), with particular reference to the following points:

1. Awareness that palliative care needs are common to many different diagnoses, not one specific pathology;
2. Requirement of early identification, to ensure a gradual and appropriate response to palliative care needs also in association with interventions aimed at prolonging life;
3. Importance of extending palliative care basic skills to the entire hospital setting.

The mission of PCS is three-pronged:

1. patient assistance by performing inpatient and outpatient consultations
2. research activities in PC
3. specialized training to improve PC core skills in health care professionals.

The PCSs have acquired advanced skills that they can transmit to other hospital professionals, according to the model from the 2nd level (specialist) to the 1st level (non-specialist) professional (28). Educational activities are offered to hospital healthcare professionals and students with the aim to improve both the quality of patient care in their specific ward and the medical education that novice professionals receive.

The educational programs proposed to professionals (29–31) belong to research projects that evaluate qualitative and/or quantitative aspects and effects on the professionals themselves. Currently, the main three lines of research of our PCS concern studies on the outcomes of palliative care education, new integrated models of assistance and evaluation of palliative care needs in patients with chronic progressive illnesses.

## Data Collection

Retrospective data regarding adults with advanced stage diseases (at least 18 years old at time of initial consultation) refers to the hospital PCS of Reggio Emilia from January 2015 to December 2019.

Data for the study has been extracted from the Local Health Service Clinical Data Repository (CDR) by the Clinical ICT Data Management unit. The CDR system gathers structured data from all clinical systems in use in the Reggio Emilia Local Health Authority in real time. The following data was extracted and processed for the study: date of death, date of admission, discharge and transfer (ADT), discharge information based on International Classification of Diseases Ninth Revision (ICD-9), E-Prescriptions and Administration information.

The data of prescription volumes of specific drugs were processed from database of the Computerized Prescription and Administration Program. All data are matched by means of a common patient identification code and a common patient contact identification code. For data protection regulatory purposes, all data extractions have been authorized and regularly traced and logged.

The study was conducted according to the guidelines of the Declaration of Helsinki and approved by Ethic Committee AVEN (Protocol UCP ITA 2017 n. 81/2017).

## Results

### Clinical Activity

In analyzing data of the clinical activity carried out for patients with palliative care needs, from January 2015 to December 2019, the PCS performed 2422 initial consultations, as shown in **Table 1**, with an average of 484 initial consultations per year.

**Table 1:** Initial consultation from January 2015 to December 2019 and comparison between initial consultation of patients with oncological disease and patients with non-oncological disease.

YEAR OF ACTIVITY	TOTAL INITIAL CONSULTATIONS	INITIAL CONSULTATIONS OF PATIENTS WITH ONCOLOGICAL DISEASE	PERCENTAGE OF PATIENTS WITH ONCOLOGICICAL DISEASE
2015	441	375	85.03%
2016	495	431	87.07%
2017	505	420	83.16%
2018	443	363	81.94%
2019	538	388	72.11%
**Total from 2015 to 2019**	**2422**	**1977**	81.62%

A majority of patients had advanced cancer as primary diagnosis and were cared for at the Azienda USL-IRCCS Hospital in Reggio Emilia. However, as shown in **Table 1**, consultations were also given to patients with advanced non-oncological diseases, for example chronic renal disease or sclerosis lateral amyotrophic, showing a decrease in percentage of oncologic consultation from 85% to 72% of total consultations. From January 2015 to December 2019, the total consultations carried out by the PCS - in particular initial consultation, control visits and family conferences - were 12,917, with an average of 2583 consultations per year and an average of 4.63 consultations per patient. An important activity of the PCS is the family conference and 1373 of them were carried out from January 2015 to December 2019. Family conferences between the patient, their family and HPs are undertaken for multiple purposes, including the sharing of information and concerns, clarifying the goals of care, discussing diagnosis, treatment, prognosis and developing a plan of care for the patient and family carers (32).

**Table 2** analyzes the number of days that patients remained in charge at the PCS from January 2015 to December 2019. During the years of activity of PCS, outpatient and inpatients remained in charge of the PCS for several days over time with an increasing trend in the days of care.

**Table 2:** Analysis of days in charge of the PCS.

Outpatients	2015	2016	2017	2018	2019
Average days in charge	13	12	38	53	77
Minimum and maximum number of days in charge	1-1904	1-1456	1-1232	1-876	1-565
**Inpatients**	**2015**	**2016**	**2017**	**2018**	**2019**
Average days in charge	8	8.5	12	18	20
Minimum and maximum number of days in charge	1-46	1-760	1-639	1-632	1-321

In the four years of activity analyzed, from 59% to 65% of cancer patients who were examined at least once by the PCS underwent CT or RT treatments at the time of the initial consultation.

The hospital was the place of death for 37 to 41% of patients who received at least one consultation from the PCS. The average number of days from admission to first consultation of the Palliative Care Service is 12 and the average number of days from first consultation or family conference to discharge is 10.

**Figure 1:**
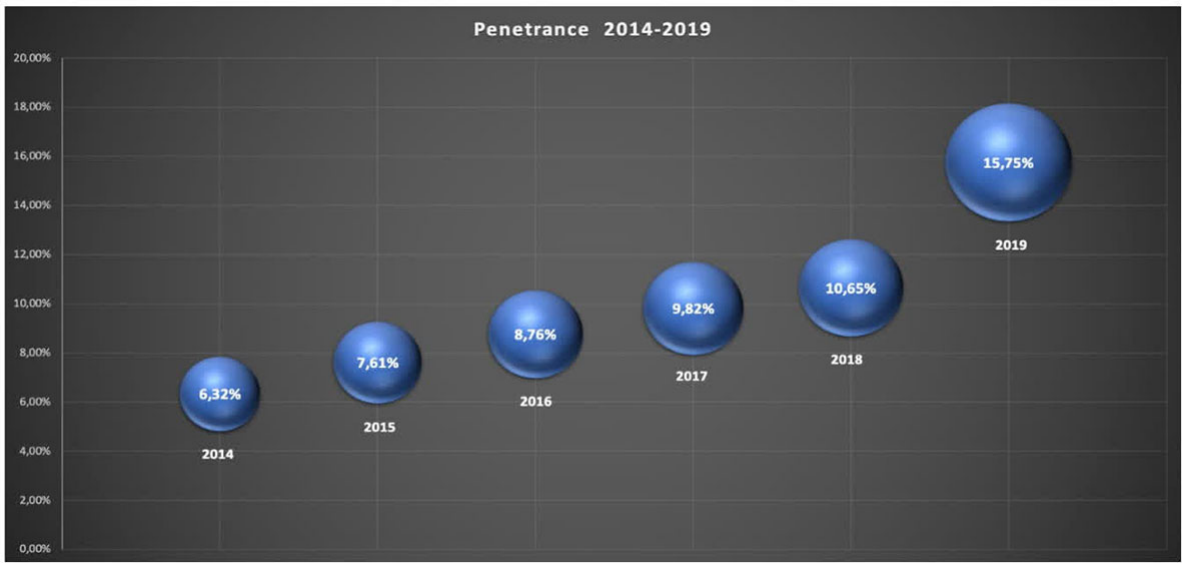
Penetrance: the percentage of patients assessed by the PCS out of the total number of cancer patients hospitalized per year.

The penetrance, that is the percentage of cancer patients assessed by the PCS out of the total number of cancer patients hospitalized per year, has increased over time from 6.3% to 15.75% (**Figure 1**).

We also evaluated the impact of the specialist palliative care service on the use of some drugs for pain management in patients with palliative care needs.

**Figure 2:**
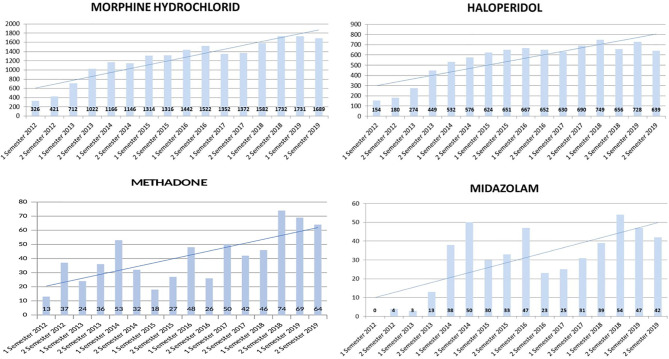
Prescription volumes of morphine, haloperidol, methadone and midazolam.

In particular, we evaluated the prescription volumes of specific opioid drugs: morphine hydrochloride, methadone and specific drugs that are used in controlling end-of-life symptoms: haloperidol and subcutaneous or hypodermic midazolam in continuous infusion within the last 72 hours of life (**Figure 2**).

**Figure 3:**
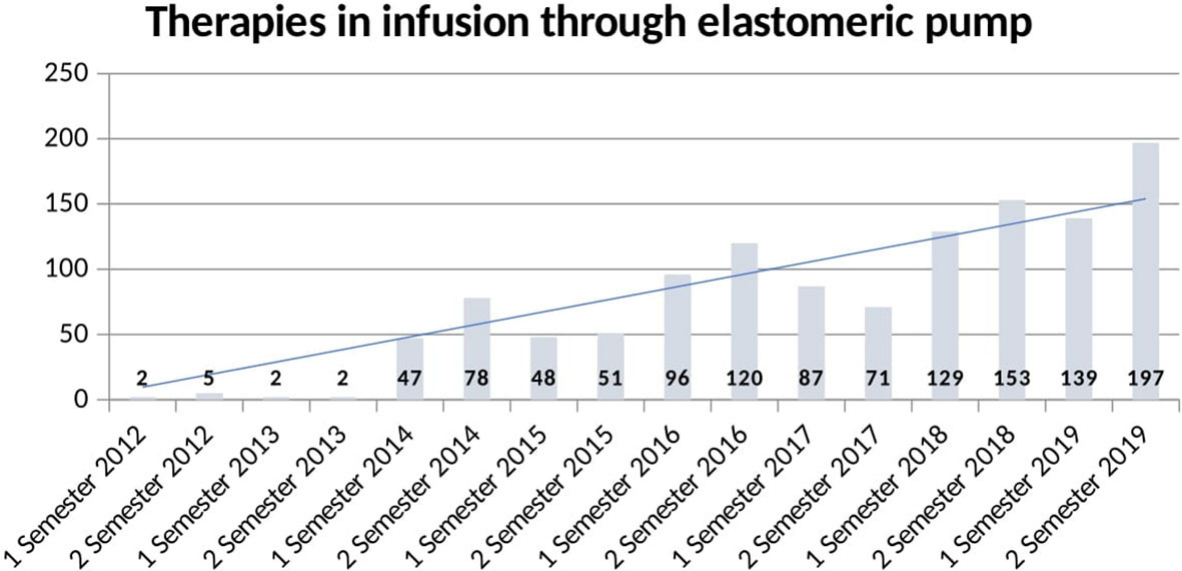
Volume of therapies administered in infusion through elastomeric pump.

Moreover, we analyzed the prescription volume of therapies administered hypodermically and in infusion through elastomeric pump (**Figure 3**).

### Educational and Research Activity

Since 2015, PCS has provided training to health professionals (HPs) of different departments of our hospital. Most of the educational projects for HPs were part of research projects, for example the communication training program performed for the Medical Oncology and Hematology Departments (33), projects carried out to improve the management of pain and end-of-life symptoms (31), and the training program, for PC-based skills for HPs from the Radiotherapy, Geriatrics and Nephrology/Dialysis wards (29).

Other projects have been implemented with the aim of training HPs to better recognize PC needs and to improve the appropriateness of requests for advice from PCS (30, 34).

PCS carries out, according to the needs of the ward, specific training on HPs (physicians and/or nurses) shared with the Bioethics Unit on palliative sedation or weekly meetings regarding the management of complex cases (35). The Bioethics Unit has also developed specific training on PCS professionals to advance first level ethical skills for consultancy (36).

We have been implemented research projects of early PC intervention in hospital for advanced cancer patients (24, 37) in which we used quality of life questionnaires and collected qualitative data.

We are currently developing a database of PSC activity that not only highlights the quantitative data, but that includes clinical data useful to better evaluate the outcome of our interventions, using some the Italian version of tools included in the Outcome Assessment and Complexity Collaborative Suite of Measure (OACC) (38).

## Discussion

In Italy, there are very few specialist PCSs dedicated full time to the care of hospitalized patients in public hospitals. To our knowledge, this study is the first to describe the activities and impact of this kind of specialist PCS in Italy. Even if administrative data is not amendable to fully describe the involvement of PCSs (39), it emerges that the performance of our PCS has grown over the years.

By evaluating the penetrance data with particular reference to the data of the American National Palliative Care Registry (40), our study shows how penetrance has significantly grown over time, with a penetrance quite superior to that of US hospitals with more than 500 beds. This data is in accordance with the vision of our PCS, the hospital’s health policy and the changes in palliative care needs of the population worldwide (2). These results also highlight hospital need for PC specialist not only as an organizational tool for protected discharge but also to ensure outpatient activities and clinical support at the patient’s bed in synergy with hospital wards.

We also believe that penetrance results obtained are partly due to the collaboration between PCS and hospital specialist teams. Although further studies are needed to validate PC penetration rates, a recent study by Gruhler 2017 (41) suggests that PC penetration rates could range from 17.6 to 26.4% in the total inpatient population. In our opinion, these positive results are linked to the continuous work of consultancy and sharing palliative care issues with other professionals, and also to specific programs of basic training on palliative care performed by the PCS in each department of our hospital (29–31).

Several strategies to manage and improve the penetration rate are already established by our service and we plan to proceed to implement these programs: we are carrying out research projects on the training of other professionals in diagnostic, therapeutic and assistance palliative care pathways and on better stratifying palliative care needs in different degrees of complexity. The aim of these projects is to improve the appropriateness of referrals to our specialized PCS. Some authors underline the importance of activating training courses to improve 1st-level skills among non-specialized PC professionals on basic principles of palliative care and to implement cooperation between the 1st and 2nd levels (28, 42, 43).

Hospital HPs recognize the expertise of PCS and the required specific training on specialized PC skills such as advance communication, treatment of pain, and palliative sedation. As in other published experiences (44), team-based learning supports the transferability of knowledge to clinical practice and the need for continuing PC training. The experience gained in recent years and the collaboration between PCS and the department has stimulated a rapid collaboration during the COVID-19 pandemic with the department at the forefront against the pandemic (30).

Over the five years analyzed, there was an increase of days in charge by PCS and number of first examinations of patients who were receiving active anticancer treatment. The greater knowledge of PCS by HPs, the educational and research project carried out and the consultations in simultaneous care have contributed to greater development of early palliative care.

Outpatient days in charge from 2015 to 2019 show an increasing rate with an average of patients in charge of more than 2 months. These results, in our opinion, could be related to a better knowledge of the PC Service by other professionals and a positive secondary outcome of the development of specific research projects (24, 29, 33).

Since the beginning of activity of the PCS, the volume of prescriptions of some drugs, such as opioids, haloperidol and midazolam, have increased in our hospital, and we also observed an increase in use of subcutaneous route for the administration of these drugs. The PCS educated hospital professionals on how to treat patients with pain and delirium and, when indicated, to manage palliative sedation with our support. Opioid analgesia is the recommended treatment for moderate to severe pain, the prevalence of which is estimated to be between 62% and 86% in advanced cancer patients (45).

Guidelines on cancer pain treatment suggest morphine as the drug of choice for the opioid analgesia (45); our data show an increase in the use of morphine.

The data collected seem to suggest that both intense training on pain management (31) and discussion on complex clinical cases concerning the use of palliative sedation contributed to the increased use of these drugs.

Published guidelines from the European Association for Palliative Care (EAPC) (46) on the use of opioid analgesics for the treatment of cancer pain recommend limiting the use of methadone to highly experienced teams because of its long, unpredictable half-life and substantial inter-individual variability of its metabolism in the liver. For this reason, since 2016 we have been carrying out specific training to all the healthcare staff of the oncology department regarding the administration of methadone and the management of its side effects, stressing the importance of supervision by the PCS.

In analyzing our data, several critical issues have emerged: the time taken to involve PCS from the beginning of hospitalization is still too long (12 days average). The data regarding the time taken to involve PCS is higher compared to that reported in the National Palliative Care Registry in the USA (40) and in a Canadian document regarding hospitals with more than 500 beds (47). A recent Danish study investigated the association between palliative care team consultation and the content and costs of hospital care in patients with advanced cancer, and the average time between hospital admission and palliative care consultation was 4 days (19).

Other critical issues are the high percentage (38%) of patients with palliative care needs who died in the hospital and a time lapse between consultancy and discharge of 10 days.

This critical issue, which in US hospitals is associated with the staffing level of PCS (22, 48), in our local reality could be due to a still insufficient early recognition of PC needs in some wards, which leads to a late consultation of the specialist team on fragile patients with poor prognosis.

Italian data shows the percentage of hospital deaths related to all chronic pathologies to settle at about 40%, while lately there has been a small decrease in cancer-related hospital deaths to approximately 35% (49). Our data confirms that the hospital is a very common place of death for cancer patients, and the importance of ensuring the delivery of quality palliative care to hospitalized patients within the activity of palliative care networks in Italy.

Data relating to the high mortality of cancer patients in our hospital suggest also that an effective collaboration with home care services within the local palliative care network is still lacking; we are working to strengthen this collaboration and we are confident in future meaningful improvements. On the other hand, a recent report suggests that an early identification of PC needs could bring to a higher acute care services utilization and hospitalization (50).

Despite this high number of cancer patients who die in the hospital in Italy, unfortunately there are still difficulties in giving specific importance to hospital PCS. Until recently, at the national level and in many regions, there was no specific code to detect the activities of the PCS, which therefore did not obtain dedicated monitoring and consequent economic enhancement. This poor visibility and enhancement continue even in the region where PC development is widespread.

In our experience, it was difficult to introduce the activities of the PCS into a PC network that did not contemplate a specialized presence of PC inside the hospital. Data relating to high mortality of cancer patients in our hospital and hospital discharge times suggests the importance of improving effective collaboration with home care services, so we are working to strengthen this collaboration and we are confident in future meaningful improvements.

## Conclusion

Demonstrating the value of a PCS in public hospitals is important to guarantee the sustainability of these services within the activities of palliative care networks in Italy. Our data suggests that a specialized hospital PCS, able to provide early palliative care to patients admitted to hospitalization and outpatient departments, and continuous training support to other hospital specialists, can relatively quickly improve the level of its penetrance in hospital activities mainly if referred to cancer patients.

The expected increase in palliative care needs will force a higher level of attention given to the appropriateness of specialist palliative care interventions. Hospitals will have to face a growing number of patients with palliative care needs, and we should implement new organizational and training models to ensure that all these patients are taken care of in accordance with the level of complexity of the needs they express.

For this aim, there is a need to promote specific research programs to evaluate the efficacy of these educational interventions and to develop dedicated and specific data systems to better document the results of the activities of Hospital PCS.

## Data Availability Statement

The datasets presented in this study can be found in online repositories. The names of the repository/repositories and accession number(s) can be found in the article/supplementary material.

## Author Contributions

SA, ST and CP: contributed to the concept and design of work; CT contributed to the analysis of the data of the prescription volume of specific drug. SA, CP and ST analyzed the data and drafted the article. All authors read and approved the final manuscript.

## Funding

The authors received no financial support for the research, authorship, and/or publication of this article. This study was partially supported by Italian Ministry of Health – Ricerca Corrente”.

## Conflict of Interest

The authors declare that the research was conducted in the absence of any commercial or financial relationships that could be construed as a potential conflict of interest.

## Publisher’s Note

All claims expressed in this article are solely those of the authors and do not necessarily represent those of their affiliated organizations, or those of the publisher, the editors and the reviewers. Any product that may be evaluated in this article, or claim that may be made by its manufacturer, is not guaranteed or endorsed by the publisher.

## References

[1] Peruselli C Manfredini L Piccione T Moroni L Orsi L. Il Bisogno Di Cure Palliative. Riv It Cure Palliat (2019) 21(1):67–74.

[2] Global Atlas of Palliative Care (2020). Available at: http://www.thewhpca.org/resources/global-atlas-on-end-of-life-care.

[3] Gomez-Batiste X Connors S. “Building Integrated Palliative Care Programs and Services”. In: Palliative-Care-Programsand-Services.-2017-B.Pdf. Liberduplex, Catalonia (2017). Available at: http://kehpca.org/wp-content/uploads/Gómez-Batiste-X-ConnorS-Eds.-Building-Integrated.

[4] Morin L Aubry R Frova L MacLeod R. Wilson DM Loucka M. Estimating the Need for Palliative Care at the Population Level: A Cross-National Study in 12 Countries. Palliat Med (2017) 31:526–36. doi: 10.1177/0269216316671280 27683475

[5] WPCA-WHO. Global Atlas of Palliative Care at the End of Life (2014). Available at: www.who.int/nmh/Global_Atlas_of_Palliative_Care.pdf.

[6] Murtagh FE Bausewein C Verne J Groenveld EI Kaloki YE Higginson IJ. How Many People Need Palliative Care? A Study Developing and Comparing Methods for Population-Based Estimates. Palliat Med (2014) 28:49–58. doi: 10.1177/0269216313489367 23695827

[7] Gomez-Batiste X Martínez-Muñoz M Blay C Espinosa J Contel JC Ledesma A. Identifying Needs and Improving Palliative Care of Chronically Ill Patients: A Community-Orientated, Population-Based, Public Health Approach. Curr Opin Support Palliat Care (2012) 12:371–8. doi: 10.1097/SPC.0b013e328356aaed 22801465

[8] Etkind SN Bone AE Gomes B Lovell N Evans CJ Higginson IJ. How Many People Will Need Palliative Care in 2040? Past Trends, Future Projections and Implications for Services. BMC Med (2017) 15:102. doi: 10.1186/s12916-017-0860-2 28514961 PMC5436458

[9] Aldridge MD Bradley EH. Epidemiology and Patterns of Care at the End of Life: Rising Complexity, Shifts in Care Patterns and Sites of Death. Health Affairs (2017) 36(7):1175–83. doi: 10.1377/hlthaff.2017.0182 28679803

[10] Arias-Casais N Garralda E Rhee J De Lima L Pons Izquierdo J Clark D. EAPC Atlas of Palliative Care (2019). Available at: https://eapcnet.wordpress.com/2019/05/24/new-edition-of-eapc-atlas-of-palliative-care-in-europe-launches-at-16th-eapc-world-congress-in-berlin/.

[11] Bekelman JE Halpern SD Blankart CR Bynum JP Cohen J Fowler R. Comparison of Site of Death, Health Care Utilization, and Hospital Expenditures for Patients Dying With Cancer in 7 Developed Countries. JAMA (2016) 315(3):272–83. doi: 10.1001/jama.2015.18603 26784775

[12] Available at: http://www.progettodemetra.it/images/ministero/DOC_WORKSHOP/5_2_SLIDE_12_12_2019_TAVOLA_ROTONDA_1.pdf.

[13] Italy. Law No. 38 (2010). Available at: http://www.salute.gov.it/portale/temi/p2_6.jsp?lingua=italiano&id=3755&area=curePalliativeTerapiaDolore&menu=legge.

[14] Shoenherr LA Bischoff KE Marks AK O'Riordan DL Patilat S. Trends in Hospital Based Specialty Palliative Care in the United States From 2013 to 2017. JAMA Network Open (2019) 2(12):e1917043. doi: 10.1001/jamanetworkopen.2019.17043 31808926 PMC6902777

[15] Centeno C Lynch T Garralda E Carrasco JM Guillen-Grima F Clark D. Coverage and Development of Specialist Palliative Care Services Across the World Health Organization European Region (2005-2012): Results From European Association for Palliative Care Task Force Survey of 53 Countries. Palliat Med (2016) 30:351–62. doi: 10.1177/0269216315598671 PMC4800456 26231421

[16] Dumanovsky T Augustin R Rogers M Lettang K Meier DE Morrison RS. The Growth of Palliative Care in U.S. Hospitals: A Status Report. J Palliat Med (2016) 19:8–15. doi: 10.1089/jpm.2015.0351 26417923 PMC4692111

[17] Agenzia Nazionale Per I Servizi Sanitari Regionali “Istruttoria Sullo Stato Di Attuazione Della Legge 38/2010 in Materia Di Rete Delle Cure Palliative”. Available at: http://servizi.agenas.it/.

[18] Italy. Report to the Italian Parliament on “Provisions to Ensure Access to Palliative Care and Pain Therapy” (2018). Available at: http://www.salute.gov.it/imgs/C_17_publication_2814_.

[19] Brinkman-Stoppelenbur A Polinder S Olij BF van denBerg B Gunnink N Hendriks MP. The Association Between Palliative Care Team Consultation and Hospital Costs for Patients With Advanced Cancer: An Observational Study in 12 Dutch Hospitals. Eur J Cancer Care (2019) 00:e13198. doi: 10.1111/ecc.13198 PMC7319483 31825156

[20] Zimmerman C Ryan S Hannon B Saltman A Rodin G Mak E. Team-Based Outpatient Early Palliative Care: A Complex Cancer Intervention. BMJ Support Palliat Care (2019) 0:1–10. doi: 10.1136/bmjspcare-2019-001903 31406013

[21] Bakitas M Lyons KD Hegel MT Lyons DK Hull JG Li Z. Effects of a Palliative Care Intervention on Clinical Outcomes in Patients With Advanced Cancer: The Project ENABLE II Randomized Controlled Trial. JAMA (2009) 302:741–9. doi: 10.1001/jama.2009.1198 PMC3657724 19690306

[22] Dumanovsky T Rogers M Spragens LH Morrison RS Meier DE. Impact of Staffing on Access to Palliative Care in U.S. Hospitals. J Pall. Med (2015) 18(12):998–9. doi: 10.1089/jpm.2015.0436 PMC4677507 26556657

[23] May P Normand C Casssel JB Del Fabbro E Fine RL Menz R. Economics of Palliative Care for Hospitalized Adults With Serious Illness: A Meta-Analysis. JAMA Internal Med (2018) 178(6):820–9. doi: 10.1001/jamainternmed.2018.0750 PMC6145747 29710177

[24] Costantini M Apolone G Tanzi S Falco F Rondini E Guberti M Fanello S. Is Early Integration of Palliative Care Feasible and Acceptable for Advanced Respiratory and Gastrointestinal Cancer Patients? A Phase 2 Mixed-Methods Study. Palliat Med (2018) 32(1):46–58. doi: 10.1177/0269216317731571 28952881

[25] Autelitano C Bertocchi E Artioli G Alquati S Tanzi S. The Specialist Palliative Care Nurses in an Italian Hospital: Role, Competence and Activities. Acta BioMed Health Prof (2021) 92(2):1–11. doi: 10.23750/abm.v92iS2.11.360 PMC8138805 33855987

[26] Comoretto N Centeno C. Experiences in Clinical Ethics: A Project for Meetings on Clinical Ethics in Palliative Medicine. Pers Bioét (2016) 20(1):38–47. doi: 10.5294/pebi.2016.20.1.4

[27] Sepúlveda C Marlin A Yoshida T Ullrich A. Palliative Care: The World Health Organization’s Global Perspective. J Pain Symptom Manage (2002) 24(2):91–6. doi: 10.1016/S0885-3924(02)00440-2 12231124

[28] Quill TE Abernethy AP. Generalist Plus Specialist Palliative Care–Creating a More Sustainable Model. N Engl J Med (2013) 368:1173–5. doi: 10.1056/NEJMp1215620 23465068

[29] Artioli G Bedini G Bertocchi E Ghirotto L Cavuto S Costantini M. Palliative Care Training Addressed to Hospital Healthcare Professionals by Palliative Care Specialist: A Mixed-Method Evaluation. BMC Palliative Care (2019) 18:88. doi: 10.1186/s12904-019-0476-8 31655585 PMC6815393

[30] Tanzi S Alquati S Martucci G De Panfilis L. Learning a Palliative Care Approach During the COVID 19 Pandemic: A Case Study in an Infectious Disease Unit. Palliat Med (2020) 18:88. doi: 10.1177/0269216320947289 32736486

[31] Tanzi S Di Leo S Mazzini E Castagnetti M Turrà C Peruselli C. Long-Term Sustainability of Quality Improvement Program on Cancer Pain Management: A Complex Intervention in an Inpatient Setting. Tumori J (2019) 106(1):25–32. doi: 10.1177/0300891619869513 31456509

[32] Hudson P Quinn K O’Hanlon B Aranda S. Family Meeting in Palliative Care: Multidisciplinary Clinical Practice Guidelines. BMC Palliat Care (2008) 7:12. doi: 10.1186/1472-684X-7-12 18710576 PMC2542352

[33] Tanzi S Luminari S Cavuto S Turola E Ghirotto L Costatini M. Early Palliative Care Versus Standard Care in Hematologic Cancer Patients at Their Last Active Treatment: Study Protocol of a Feasibility Trial. BMC Palliat Care (2020) 19(1):53. doi: 10.1186/s12904-020-00562-w 32321483 PMC7178743

[34] Tanzi S Martucci G Autelitano C Alquati S Peruselli A Artioli G. Consultations’ Demand for a Hospital Palliative Care Unit: How to Increase Appropriateness? Implementing and Evaluating a Multicomponent Educational Intervention Aimed at Increase Palliative Care Complexity Perception Skill. BMC Palliative Care (2022) 21:90. doi: 10.1186/s12904-022-00968-7 35619110 PMC9133822

[35] De Panfilis L Peruselli C Artioli G Perin M Bruera E Brazil K. A Qualitative Study on Nudging and Palliative Care: An Attractive But Misleading Concept. Int J Environ Res Public Health (2021) 18(18):1–8. doi: 10.3390/ijerph18189575 PMC8468152 34574501

[36] De Panfilis L Tanzi S Perin M Turola E Artioli G. Teach for Ethics in Palliative Care: A Mixed-Method Evaluation of a Medical Ethics Training Programme. BMC Palliat Care (2020) 19:149. doi: 10.1186/s12904-020-00653-7 32977796 PMC7519533

[37] Maltoni M Scarpi E Dall’Agata M Schiavon S Biasini C Codecà C. Systematic Versus on-Demand Early Palliative Care: A Randomized Clinical Trial Assessing Quality of Care and Treatment Aggressiveness Near the End of Life”. Eur J Cancer (2016) 69:110–8. doi: 10.1016/j.ejca.2016.10.004 27821313

[38] Veronese S. La Versione Italiana Di OACC: Dalla Valutazione Dei Bisogni Alla Pianificazione Delle Cure Palliative. Riv It Cure Palliat (2020) 22(3):147–58.

[39] Stubbs JM Hassareh H Achat HM Greenaway S Muruganantham P. Specialist Palliative Care Activity at an Acute Tertiary Hospital and its Representation in Administrative Data. Am J Hospice Palliat Med (2020) 38(3):216–22. doi: 10.1177/10499091120939861 32662294

[40] Rogers M Dumanovsky T. National Landscape of Hospital-Based Palliative Care: Findings From the National Palliative Registry. Natl Palliative Care Registry (2017). doi: 10.1016/j.jpainsymman.2016.12.233

[41] Gruhler H Krutka A Luetke-Stahlman H Gardner E. Determining Palliative Care Penetration Rates in Acute Care Setting. J Pain Symptom Manage (2018) 55(2):226–35. doi: 10.1016/j.jpainsymman.2017.09.013 28935130

[42] Costantini M. Sfide E Opportunità Delle Cure Palliative Moderne. Bologna: ASMEPA Edizioni (2017).

[43] Block SD Billings JA. A Need for Scalable Outpatient Palliative Care Interventions. Lancet (2014) 383:1699–700. doi: 10.1016/S0140-6736(13)62676-8 24559580

[44] Ballangrud R Aase K Vifladt A. Longitudinal Team Training Program in a Norwegian Surgical Ward: A Qualitative Study of Nurses' and Physicians' Experiences With Implementation. BMC Health Serv Res (2021) 21:725. doi: 10.1186/s12913-021-06732-6 34294085 PMC8299676

[45] WHO Guidelines for the Pharmacological and Radiotherapeutic Management of Cancer Pain in Adults and Adolescents. Geneva: World Health Organization (2018). 30776210

[46] Caraceni A Hanks G Kaasa S Bennett MI Brunelli C Cherny N. Use of Opioid Analgesics in the Treatment of Cancer Pain: Evidence Based Recommendations From the EAPC. Lancet Oncol (2012) 13:e58–68. doi: 10.1016/S1470-2045(12)70040-2 22300860

[47] Canadian Institute for Health Information. “Access to Palliative Care in Canada”. Available at: https://www.cihi.ca/sites/default/files/document/access-palliative-care-2018-en-web.pdf.

[48] Spetz J Dudley N Trupin L Rogers M Meier DE Dumanovsky T. Few Hospital Palliative Care Programs Meet National Staffing Recommendations. Health Affairs (2016) 35:1690–97. doi: 10.1377/hlthaff.2016.0113 27605652

[49] Italian Ministry of Health . Available at: http://www.salute.gov.it/imgs/C_17_pubblicazioni_2814_allegato.pdf.

[50] Mittman N Liu N MacKinnon M Seung SJ Look Hong NJ Earle CC. Does Early Palliative Identification Improve the Use of Palliative Care Services? PLoS One (2020) 15(1):e022659. doi: 10.1371/journal.pone.0226597 PMC6994244 32005036

